# Evaluation and analysis of municipal solid wastes in Tehran, Iran

**DOI:** 10.1016/j.mex.2018.04.003

**Published:** 2018-04-10

**Authors:** Emad Dehghanifard, Mohammad Hadi Dehghani

**Affiliations:** a Department of Environmental Health Engineering, School of Public Health, Alborz University of Medical Sciences, Karaj, Iran; b Research Center for Health, Safety & Environment (HSE), Alborz University of Medical Sciences, Karaj, Iran; c Department of Environmental Health Engineering, School of Public Health, Tehran University of Medical Sciences, Tehran, Iran; d Institute for Environmental research, Center for Solid Waste Research, Tehran University of Medical Sciences, Tehran, Iran

**Keywords:** Municipal solid waste, Segregation, Recycling, Paper, Plastic

## Abstract

Waste management for municipal solid waste is considered a public health services, providing citizens with a system of disposing of their waste in an environmentally sound and economically feasible way. The amount and composition of waste generated comprise the basic information needed for the planning, operation and optimization of waste management systems. In this study, paper and plastic quantity changes in the MSW (municipal solid waste) of the region 10 of Tehran city were evaluated. This study was conducted in 6 months, in the summer and autumn seasons, at the region 10 of Tehran city. In this study, paper and plastic were segregated and data were analyzed by SPSS software. The paper parameter of solid wastes was consisted of folder paper, cardboard and used newspaper. The plastic parameter of solid wastes was consisted of plastic materials, plastic house shoes, plastic sack, nylon sack, linoleum, radiology photograph and PET. Samples were collected and weighed daily. The total quantity of paper and plastic portion of this region solid waste were 203,930 Kg and 180,101 Kg, respectively. The percentage of paper and plastic portion of this region solid waste also were 6.82% and 6.03%, respectively. The analyses showed a significant difference between these parameters and season, some months and days (P-value < 0.05). So, because of the results of this study and economical issues, the paper and plastic segregation from generated source point and recycling them are important.

## Graphical abstract

**Figure:**
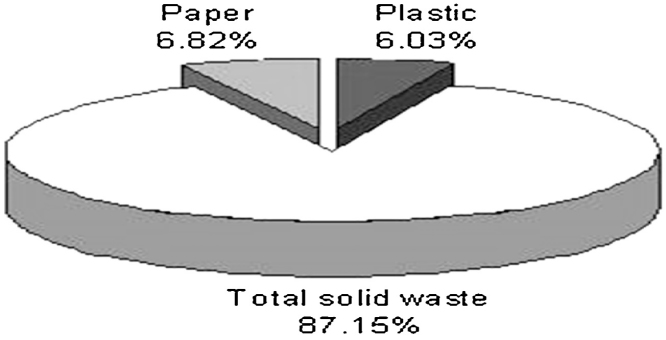

## Introduction

**Fig. 1:**
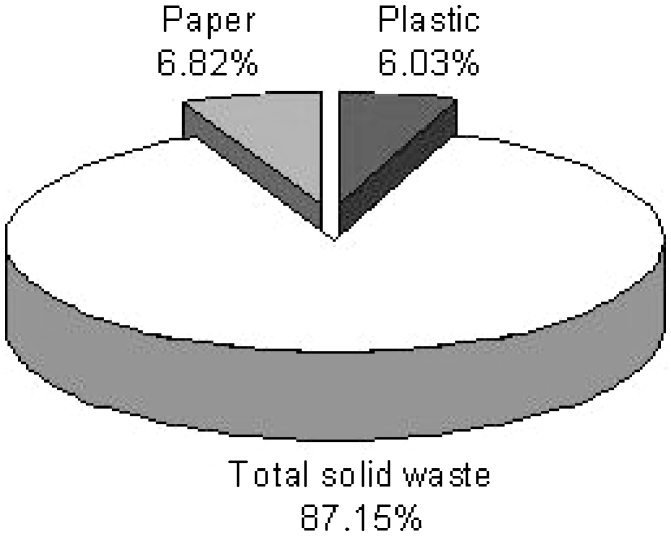
The paper and plastic percentage in total solid waste.

**Fig. 2:**
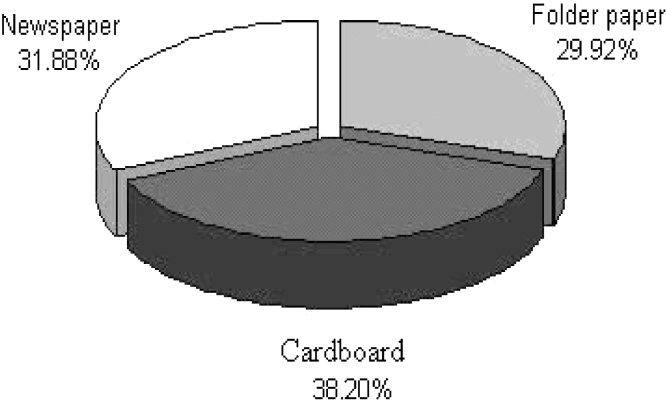
The percentage of paper components.

**Fig. 3:**
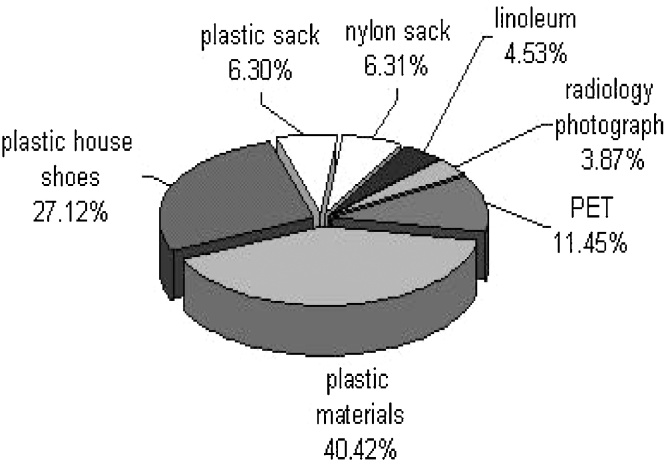
The percentage of plastic components.

The cornerstone of successful planning for a waste management program is the availability of reliable information about the quantity and the type of material being generated and an understanding about how much of that material that collection program manager can expect to prevent or capture. Effective waste management through MSW composition studies is important for numerous reasons, including the need to estimate material recovery potential, to identify sources of component generation, to facilitate design of processing equipment, to estimate physical, chemical, and thermal properties of the waste and to maintain compliance with national law and European directives. The composition of generated waste is extremely variable as a consequence of seasonal, lifestyle, culture, demographic, geographic, and legislation impacts. This variability makes defining and measuring the composition of waste more difficult and at the same time more essential [[1], [2], [3]] (Fig. 1, Fig. 2, Fig. 3)

The region 10 is on of the most crowded regions of Tehran city. The Area of this region is about 807 ha (8,070,000 m^2^) and is restricted from north by Azadi Ave., from south by Qazvin Ave., from east by Navab highway and from west by Shahidan St.

According to Waste Framework Directive 75/442/EEC [4], member states are obliged to take the necessary measures in order to ensure that the final disposal or exploitation of waste is performed without endangering public health and without causing any damage to the environment. In its 1996 Communication on the Review of the Community Strategy for Waste Management1 the EU Commission enacted the waste management hierarchy: prevention/minimization, reuse, recycling, energy recovery and safe disposal. It should be pointed out that this hierarchy is just a proposal and not a result of a detailed environmental and economic evaluation of each alternative. Such an evaluation has to be performed so that the regional solid waste authorities are able to choose the appropriate management scheme.

The primary goal of this study was to develop a representative, statistically defensible estimation of the waste composition for the region 10 of Tehran city. The final goal was determining the quantity and quality of segregated solid wastes for recycling management in this region.

## Methodology

### Waste collection

This study was conducted in 6 months of summer and autumn seasons. In this study, paper and plastic were considered. The paper consisted of folder paper, cardboard and used newspaper. The plastic consisted of plastic materials, plastic house shoes, plastic sack, nylon sack, plastic linoleum, radiology photograph and PET. The habitants of this region were instructed to segregate their solid waste to solid and wet parts and hold them in separate sacks, daily. Then, city hall dustmen collected, carried and numerated solid waste sacks daily from homes to the Tehran recycling station. Segregated waste sacks were first reopened, and then weighed through digital weight scale. The participation rate of habitants was estimated to 70%.

### Waste analyzing

Obtained data were analyzed by SPSS software for determining the significant difference between the paper and plastic parts and considered seasons, months and days, as mentioned above. Several statistical methods were used in this study. First, the normal condition of the data was determined by the Kolmogorov-Smirnov test (P-value < 0.05). Then, for normal parts of those parameters, the One-way ANOVA test and Independent-sample T-test and for abnormal parameters, the Mann-Whitney and Kruskal-Wallis Tests were used. Then, the results were compared with other results of similar studies in Iran and other parts of the world.

## Results and discussion

### Normal condition analyzing

The normal condition of the data was determined by the Kolmogorov-Smirnov test (P-value < 0.05). This test showed that plastic materials and plastic house shoes were normal and other components were abnormal.

### Comparing solid waste amounts in different seasons

Table 1 shows the mean and S.D (standard deviation) of the paper and plastic components in the summer and autumn seasons. Using the One-way ANOVA test for comparing the mean of plastic materials and plastic house shoes with seasons showed that there was a significant difference between these parameters (P-value < 0.001). Also, by using the Mann-Whitney test for the abnormal solid waste data and the seasons, there was a significant difference for cardboard, used newspaper, plastic sack, nylon sack, plastic linoleum, radiology photograph and PET, but there was no significant difference for folder paper (P-value < 0.05).

**Table 1:** The mean and S.D of the paper and plastic components in the summer and autumn seasons.

MSW Kinds	Component	Summer			Autumn		
		Sample No.	Mean	S.D	Sample No.	Mean	S.D
Paper	Folder paper	93	337.63	105.14	90	331.36	38.62
	Cardboard	93	404.95	71.02	90	447.17	51.88
	Newspaper	93	309.44	40.57	90	369.24	27.22
Plastic	Plastic materials	93	371.39	55.97	90	421.16	36.58
	Plastic house shoes	93	246.28	42.71	90	285.64	32.02
	Plastic sack	93	61.98	51.04	90	61.96	90
	Nylon sack	93	64.23	48.92	90	59.93	86.85
	Linoleum	93	45.19	53.92	90	44.04	74.23
	Radiology photograph	93	37.28	50.44	90	38.93	74.74
	PET	93	89.47	17.85	90	122.21	31.7

### Comparing solid waste amounts in different months

In Table 2, the mean and S.D. of paper and plastic components in certain months, are showed. The One-way ONOVA and Kruskal-Wallis tests were used for comparing the normal data means and abnormal data mean ranks with months, respectively. As represented in Table 3, the results of these tests showed that there are some significant differences between these data and certain months (P-value < 0.05).

**Table 2:** The mean and S.D of the paper and plastic components in the summer and fall months.

MSW kinds	component	June			July			August			September			October			November			December		
		Sample No.	Mean	S.D	Sample No.	Mean	S.D	Sample No.	Mean	S.D	Sample No.	Mean	S.D	Sample No.	Mean	S.D	Sample No.	Mean	S.D	Sample No.	Mean	S.D
Paper	Folder paper	9	565.89	78.93	31	329.13	101.62	31	308.1	45.84	30	301.6	49.23	31	323.45	42.47	30	331.23	38.84	21	350.62	26.89
	Cardboard	9	391.44	50.16	31	380.29	72.47	31	415.58	48.39	30	434.77	80.34	31	442.29	40.56	30	450.53	42.68	21	449.52	80.23
	Newspaper	9	286.78	20.57	31	289.94	40.24	31	320.61	38.76	30	339.67	34.91	31	360.39	23002	30	372.97	34.54	21	378.62	18.27
Plastic	Plastic materials	9	403.56	44.33	31	333.39	62.154	31	383.77	39.06	30	398.53	41.38	31	416.03	37.95	30	422.43	38.2	21	431.1	33.12
	Plastic house shoes	9	259.67	39.25	31	225.03	44.74	31	259.71	29.33	30	255.97	42.43	31	270.84	18.91	30	285.8	34.51	21	314.24	28.57
	Plastic sack	9	72.67	14.15	31	59	38.35	31	62.65	56.37	30	62.13	68.58	31	156.67	82.83	30	174.6	88.49	21	67.86	111.25
	Nylon sack	9	77	13.45	31	67.97	29.21	31	64.06	61.95	30	52.83	61.92	31	145.21	80.2	30	166.92	91.53	21	45.71	97.65
	Linoleum	9	77.33	8.62	31	62.23	38.74	31	19.55	46.58	30	37.73	70.45	31	161.9	78.23	30	165.63	75.12	21	40.95	75.44
	Radiology photograph	9	57.22	15.41	31	38.94	25.11	31	31.94	58.46	30	35.50	70.13	31	150.4	56.49	30	182.13	84.05	21	46.90	86.41
	PET	9	83.44	7.92	31	83.45	14.43	31	89.65	18.01	30	96.8	20.12	31	118.68	29.72	30	132.57	33.33	21	125.86	27.61

**Table 3:** The P-value numbers of paper and plastic component in summer and fall months.

MSW kinds Component		P-value																				
		Jun						Jul					Aug				Sep			Oct		Nov
		Jul	Aug	Sep	Oct	Nov	Dec	Aug	Sep	Oct	Nov	Dec	Sep	Oct	Nov	Dec	Oct	Nov	Dec	Nov	Dec	Dec
paper	Folder paper	0.00*	0.00*	0.00*	0.00*	0.00*	0.00*	0.89	0.00*	0.35	0.10	0.01*	0.60	0.20	0.08	0.00*	0.00*	0.02*	0.00*	0.45	0.02*	0.03*
	Cardboard	0.70	0.31	0.09	0.00*	0.00*	0.00*	0.04*	0.01*	0.00*	0.00*	0.00*	0.33	0.02*	0.00*	0.00*	0.45	0.03*	0.00*	0.34	0.06	0.43
	Newspaper	0.80	0.01*	0.00*	0.00*	0.00*	0.00*	0.00*	0.00*	0.00*	0.00*	0.00*	0.04*	0.00*	0.00*	0.00*	0.43	0.00*	0.00*	0.06	0.00*	0.28

Plastic	Plastic materials	0.00*	0.23	0.76	0.45	0.26	0.11	0.00*	0.00*	0.00*	0.00*	0.00*	0.19	0.00*	0.00*	0.00*	0.12	0.03*	0.01*	0.57	0.22	0.49
	Plastic house shoes	0.01*	0.10	0.78	0.40	0.05	0.00*	0.00*	0.00*	0.00*	0.00*	0.00*	0.67	0.21	0.00*	0.00*	0.09	0.00*	0.00*	0.09	0.00*	0.00*
	Plastic sack	0.25	1	0.71	0.28	0.13	0.07	0.47	0.28	0.27	0.09	0.05	1	0.59	0.41	0.32	0.56	0.60	0.63	0.85	0.95	0.90
	Nylon sack	0.25	0.70	0.35	0.52	0.35	0.01*	0.93	0.93	0.41	0.17	0.00*	0.59	0.94	0.82	0.06	0.82	0.70	0.18	0.96	0.18	0.23
	Linoleum	0.10	0.00*	0.01*	0.11	0.04*	0.02*	0.00*	0.00*	0.09	0.20	0.02*	0.32	0.07	0.20	0.34	0.02*	0.70	0.83	0.73	0.66	0.88
	Radiology photograph	0.07	0.01*	0.01*	0.00*	0.04*	0.02*	0.01*	0.01*	0.00*	0.30	0.02*	0.98	0.43	0.58	0.74	0.12	0.63	0.75	0.20	0.31	0.96
	PET	0.95	0.23	0.02*	0.00*	0.00*	0.00*	0.15	0.60	0.00*	0.00*	0.00*	0.14	0.00*	0.00*	0.00*	0.09	0.00*	0.00*	0.05	0.32	0.58

* The mean difference is significant at the 0.05 level.

### Comparing solid waste amounts in different days

Table 4 showed the mean and S.D. of paper and plastic components in all days of a week. The One-way ONOVA and Mann-Whitney tests were used for comparing the normal data means and abnormal data mean ranks with days, respectively. Table 5 showed the results of these tests and those parameters that have a significant difference with certain days.

**Table 4:** The mean and S.D of the paper and plastic components in the summer and fall days.

MSW kinds	component	Mon			Tue			Wed			Thu			Fri			Sat			Sun		
		Sample No.	Mean	S.D	Sample No.	Mean	S.D	Sample No.	Mean	S.D	Sample No.	Mean	S.D	Sample No.	Mean	S.D	Sample No.	Mean	S.D	Sample No.	Mean	S.D
Paper	Folder paper	26	337.65	92.22	26	336.23	78.3	26	325.12	58.88	26	324.38	57.04	27	341.44	95.98	26	334.65	79.86	26	342.08	91.81
	Cardboard	26	428.08	57.76	26	418.81	61.98	26	417.04	60.63	26	432.88	52.72	27	450.11	83.71	26	412.15	79.18	26	419.96	56.2
	Newspaper	26	341	38.86	26	342.5	44.83	26	331.08	47.14	26	346.73	44.07	27	336.96	43.6	26	337.5	45.87	26	336.27	57.59
Plastic	Plastic materials	26	399.96	54.71	26	398.31	49.62	26	389.35	50.16	26	401.19	53.87	27	395.04	56.04	26	390.77	58.63	26	396.46	55.88
	Plastic house shoes	26	264.46	43.35	26	260.12	43.58	26	264.54	41.71	26	261.69	37.64	27	271.07	47.78	26	272.96	36.92	26	264.42	48.85
	Plastic sack	26	64.81	73.02	26	43.77	58.95	26	67.23	73.57	26	59.08	79.41	27	50.56	57.43	26	83.65	90.25	26	65.12	72.71
	Nylon sack	26	59.27	58.77	26	86.23	84.22	26	48.88	64.34	26	44.42	62.05	27	90	72.38	26	42.08	67.53	26	62.85	69.06
	Linoleum	26	63.81	72.05	26	23.92	48.06	26	58.38	72.65	26	40.77	63.35	27	51.22	66.51	26	38.54	60.26	26	35.5	63.89
	Radiology photograph	26	35.08	58.63	26	25.04	47.83	26	25.58	58.04	26	44.73	74.6	27	50.96	69.91	26	25.19	50.64	26	59.58	75.66
	PET	26	115.58	35.12	26	99.08	22.37	26	111.73	32.73	26	101.35	30.6	27	107.37	31.67	26	105	32.21	26	98.85	25.13

**Table 5:** The P-value numbers of paper and plastic component in summer and fall days.

MSW kinds Component		P-value																				
		Mon						Tue					Wed				Thu			Fri		Sat
		Tue	Wed	Thu	Fri	Sat	Sun	Wed	Thu	Fri	Sat	Sun	Thu	Fri	Sat	Sun	Fri	Sat	Sun	Sat	Sun	Sun
paper	Folder paper	1	0.90	0.96	0.88	0.87	0.90	0.75	0.84	0.81	0.88	0.90	0.94	0.94	0.90	0.90	1	0.92	0.79	0.94	0.76	0.74
	Cardboard	0.42	0.56	0.68	0.66	0.52	0.63	0.99	0.37	0.21	0.98	0.98	0.39	0.17	0.95	0.97	0.78	0.43	0.34	0.18	0.18	0.91
	Newspaper	0.78	0.52	0.52	0.70	0.75	0.73	0.40	0.85	0.59	0.59	0.52	0.34	0.71	0.67	0.86	0.37	0.51	0.49	0.91	0.96	0.91
Plastic	Plastic materials	0.91	0.48	0.93	0.74	0.81	0.54	0.55	0.85	0.83	0.62	0.90	0.43	0.70	0.92	0.64	0.68	0.49	0.75	0.77	0.92	0.71
	Plastic house shoes	0.72	0.99	0.82	0.58	0.99	0.48	0.71	0.89	0.36	0.28	0.72	0.81	0.58	0.48	0.99	0.43	0.35	0.82	0.87	0.57	0.48
	Plastic sack	0.29	0.89	0.63	0.54	0.98	0.46	0.22	0.62	0.61	0.09	0.28	0.53	0.53	0.59	0.86	0.91	0.21	0.62	0.22	0.51	0.50
	Nylon sack	0.30	0.47	0.25	0.12	0.83	0.14	0.08	0.05	0.73	0.02*	0.36	0.76	0.03*	0.55	0.37	0.01*	0.77	0.30	0.00	0.23	0.19
	Linoleum	0.03*	0.77	0.20	0.58	0.15	0.19	0.06	0.33	0.09	0.35	0.54	0.36	0.89	0.35	0.28	0.47	0.96	0.72	0.41	0.29	0.79
	Radiology photograph	0.54	0.49	0.71	0.42	0.21	0.64	0.95	0.36	0.12	0.86	0.07	0.30	0.15	0.80	0.06	0.66	0.45	0.39	0.20	0.57	0.08
	PET	0.06	0.73	0.15	0.37	0.04*	0.23	0.17	0.79	0.39	0.64	0.78	0.27	0.70	0.44	0.15	0.58	0.84	0.65	0.74	0.26	0.49

* The mean difference is significant at the 0.05 level.

### Comparing the results with similar studies

This test method is applied for the determination of the mean composition of MSW based on the collection and manual sorting of a number of samples of waste over a selected time of period covering a 183 days. This test method includes procedures for the collection of a representative sorting sample of unprocessed waste, manual sorting of the waste into individual waste components, data reduction, and reporting of the results. This test method may be applied at landfill sites, waste processing and conversion facilities, and transfer stations. Its level of confidence and precision values are, respectively, 95% and 0.05%.

The results of this study showed that the total weight of paper and plastic parameters in the solid waste of this region were 203,930 and 180101 kg, and the percentage of these parameters were 6.82%and 6.02%, respectively.

So the total weight of paper and plastic is assumed about 406,745 and 359217 kg, respectively. In comparing the previous study that conducted in 1991 on the solid waste of Tehran province that showed the amount of paper in the solid waste was 10.3% with this study results, it seems that the amount of paper was decreased during last decade [5]. In 1995, a study was conducted on the MSW of Ahvaz province and the results showed that the percentage of paper and plastic were 11.3% and 4.26%, respectively [6]. In 1996, the results of a study that was conducted on the MSW of Mazandaran province showed that the percentage of paper was 7.14% [7]. In 2000, a study was conducted On MSW of Hamedan city and the paper and plastic percentage were 5.75% and 7.7%, respectively [8]. In 2000, another study was conducted on Sabzevar city MSW and the results showed that the percentage of paper and plastic were 4.9% and 6.46%, respectively [9]. Results of a study that conducted on MSW of Yazd city in 2001 showed that the percentage of paper in that MSW was 5.57% [10]. In 2001, another study was conducted on MSW of Hamedan city and the study showed that the paper percentage was 9.82% [11]. In 2002, a study was conducted on MSW of Kashan city villages and results showed that the paper and plastic percentage were 1.48% and 1.51%, respectively [12]. In 2002, a study was conducted on the municipal solid waste system and solid waste characterization at the municipality of Veles, Macedonia and the paper and plastic percentage were 24.47% and 7%, respectively [13]. In 2002, another study was conducted on MSW of Metro manila, Philippines and the paper and plastic percentage were 29% and 28%, respectively [14]. In 2006, a study in the Crete, Greece showed that the percentage of paper and plastic in MSW were 19.94 and 16.85, respectively [15]. In comparing the results of those studies with this study, it seems that the amount of plastic in urban areas of Iran has been increased but the amount of paper has been decreased or stabled. Also, it seems that the amount of paper and plastic in rural areas of Iran are much lower than urban areas. This is a serious problem in Iran cities because the amount of plastic (hardly recyclable materials) has been increased and recycling them are harder than paper. Iran government tries should be on decreasing plastic usage in urban areas and increase the paper or other organic recyclable usage, instead. As mentioned above, the amount of paper and plastic in foreign areas are much more than areas in Iran. But the relation between paper and plastic is greater than 1 in those areas and it seems that they have less problem about recycling the paper and plastic.

## Conclusions

The Iranian recycling and material recovery organization reported that the income of each kilogram paper and plastic recycling is about 2.5 and 6.5 cents, respectively. By calculating these numbers in the total collected amount of paper and plastic parameters in this region, the total yearly income of paper and plastic recycling is about 10,162 and 23,350 dollars, respectively. So, the special notice from economical point of view to the amount of paper and plastic in the MSW of this region and recycling them is very critical. Unfortunately, the most of MSW of this region is buried without any recycling process and the Tehran municipality organization should make instant and effective decision to recycling the recyclable materials that buried daily.
